# Account of Diseases in an Eastern District of London, from the 20th of February to the 20th of March, 1800

**Published:** 1800-04

**Authors:** 


### STATE OF DISEASES IN LONDON.

*Account of Difeafes in an Eaftern Diftrict of London, from the 20th of February to the 20th of March,* 1800.

| | No. of Cafes. |
|---|---|
| ACUTE DISEASES. | |
| Pleuritis | 2 |
| Peripneumony | 4 |
| Peripneumonia Notha | 6 |
| Acute Rheumatifm | 1 |
| CHRONIC DISEASES. | |
| Cough | 16 |
| Dyfpnœa | 7 |
| Cough with Dyfpnœa | 19 |
| Raucedo | 2 |
| Hæmoptoe | 4 |
| Hydrothorax | 2 |
| Pleurodyne | 3 |
| Dyfpepfia | 5 |
| Vomitus | 4 |
| Gaftrodynia | 6 |
| Hypochondriafis | 2 |
| Colica | 2 |
| Diarrhœa | 15 |

Hæmorrhois

| | No. of Cafes. | | No. of Cafes. |
|---|---|---|---|
| Hæmorrhois | 3 | Chronic Rheumatifm | 18 |
| Dyfuria | 2 | PUERPERAL DISEASES. | |
| Dolor Nephriticus | 1 | Ifchuria | 1 |
| Amenorrhœa | 6 | Rhagas Papillæ | 3 |
| Fluor Albus | 5 | Menorrhagia lochialis | 3 |
| Anafarca | 3 | Maftrodynia | 4 |
| Vertigo | 4 | INFANTILE DISEASES. | |
| Hyfteria | 5 | Fever | 1 |
| Epilepfia | 1 | Meafles | 4 |
| Ophthalmia | 3 | Ophthalmia | 3 |
| Otalgia | 2 | Convulfio | 3 |
| Rheumatifmus Odontalgicus | 4 | Diarrhœa | 5 |

The long continuance of north and north-eafterly winds, has protracted the duration of thofe difeafes, which are moft frequent in the early months of winter, to a later period. Difeafes of the pulmonic fyftem are ftill prevalent, and, in many cafes, very obftinate. Peripneumony, both of the true and the baftard fpecies, has been a common complaint, and, to many perfons far advanced in life, it has proved fatal. This difeafe, in feveral patients, could be traced to a very imprudent expofure of themfelves to the influence of the very cold winds, which have blown, with but little intermiffion, from the eaft, the north, or north-eaft points. During fuch a ftate of the atmofphere, the moft proper climate for old perfons and young children is a warm room.

The meafles, a difeafe of which children are moft frequently the fubjects, has been aggravated in fome of its fymptoms by the ftate of the weather. The cough and affections of the refpiratory organs have been very troublefome, and even threatening, though the difeafe has not, in any of the inftances referred to, proved fatal. Diarrhœa has been very frequent, as appears by the preceding lift, and has been often tedious in its duration. This complaint, in feveral cafes, might be referred to an expofure to cold; and has, in fome inftances, recurred upon too early a return to former habits of diet and regimen.

Rheumatifm, both of the acute and chronic fpecies, has frequently occurred during the laft few weeks. A fpecies of this difeafe, which nofologifts have denominated Rheumatifmus Odontalgicus, has been troublefome. This is principally characterized by a pain in the teeth, and in the mufcles of the face, which, inftead of being confined to one fpot, frequently changes its feat, and, like other rheumatic affections, is apt to return upon every flight occafion.

